# Agent-based Modeling for Ontology-driven Analysis of Patient Trajectories

**DOI:** 10.1007/s10916-020-01620-8

**Published:** 2020-08-02

**Authors:** Davide Calvaresi, Michael Schumacher, Jean-Paul Calbimonte

**Affiliations:** University of Applied Sciences and Arts Western Switzerland, HES-SO Valais-Wallis, TechnoPole 3, CH-3960 Sierre, Switzerland

**Keywords:** Patient trajectories, Semantic modeling, Agent-based modeling

## Abstract

Patients are often required to follow a medical treatment after discharge, e.g., for a chronic condition, rehabilitation after surgery, or for cancer survivor therapies. The need to adapt to new lifestyles, medication, and treatment routines, can produce an individual burden to the patient, who is often at home without the full support of healthcare professionals. Although technological solutions –in the form of mobile apps and wearables– have been proposed to mitigate these issues, it is essential to consider individual characteristics, preferences, and the context of a patient in order to offer personalized and effective support. The specific events and circumstances linked to an individual profile can be abstracted as a patient trajectory, which can contribute to a better understanding of the patient, her needs, and the most appropriate personalized support. Although patient trajectories have been studied for different illnesses and conditions, it remains challenging to effectively use them as the basis for data analytics methodologies in decentralized eHealth systems. In this work, we present a novel approach based on the multi-agent paradigm, considering patient trajectories as the cornerstone of a methodology for modelling eHealth support systems. In this design, semantic representations of individual treatment pathways are used in order to exchange patient-relevant information, potentially fed to AI systems for prediction and classification tasks. This paper describes the major challenges in this scope, as well as the design principles of the proposed agent-based architecture, including an example of its use through a case scenario for cancer survivors support.

## Introduction

The importance of sustained support over extended periods of time is particularly important for patients, especially for rehabilitation, chronic diseases, or other conditions such as those affecting cancer survivors. In these situations, patients are often left at home, expected to continue their lives and activities, while dealing with potential complications and issues inherent to their health conditions [27]. To support them effectively in this delicate phase, healthcare providers need to have a sufficient understanding of the individual pathways of each patient, as well as the potential risks and courses of action [19]. Each patient may respond differently to treatments, depending on a series of factors, including demographics, health conditions, psychological aspects, social and emotional characteristics, etc. Although it is undoubtedly complicated and even expensive to have such a detailed picture of each patient’s situation using traditional approaches, nowadays, the use of digital solutions for personal data monitoring and coaching opens the ways for personalized healthcare. Such solutions include the usage of artificial intelligent (AI) techniques —including machine learning (ML) based data analytics— through the exploitation of large volumes of personal health data acquired from patients going through different health pathways.

The concept of illness trajectories [31], describing the different events and situations a patient experiences through a given illness, can be broadened to what is called a patient trajectory [3]. Beyond the scope of an illness, a patient trajectory encompasses contextual data from the patient, even before diagnosis, and may include multiple co-morbidities, as well as emotional and social indicators, self-reported outcomes, and wellness monitoring observations during and after treatment [14, 41]. The usage of data analytics based on ML techniques applied to this vast body of data can provide a number of features including: patient stratification, identification of unusual behavior patterns, prediction of wellness and distress parameters, assessment of home exercise performance, improvement of adherence to treatment, identification and prevention of risk situations. On the one hand, the information contained in these trajectories requires managing and integrating (potentially) very diverse types of data, ranging from electronic health records [8, 18] to self-reported observations [20] or sensor measurements recorded by a wearable device [10]. The data *variety* and *distribution* aspects are, therefore, fundamental problems to be addressed. On the other hand, as a consequence, the management of this information requires taking into account specific concerns regarding data *distribution*, reuse conditions, sharing among different care structures, confidentiality & privacy. In particular, the agent-oriented approach characterizes the majority of assistive systems operating with distributed and heterogeneous data [12]. Agent-based systems can ensure a high-degree of personalization [4], autonomy, distributed collaborative/competitive intelligence, and security.

Therefore, in the context of patient trajectory analytics, the main high-level requirements are: to handle broad-scope information, heterogeneous data-sources, and distributed data producers and consumers. These requirements entail scientific challenges related to (i) the modeling of patient trajectories under heterogeneity constraints; and (ii) the design of decentralized digital infrastructures for analyzing and sharing these trajectories. In this paper, we propose addressing these two challenges by introducing an agent-based modeling approach that relies on the use of semantic modeling of patient trajectories. The rationale behind this design is that ontology models can effectively help to describe events and circumstances of a patient with respect to her health condition, while autonomous agents can represent her interests facing other agents, which may act on behalf of other patients, healthcare providers, and data analytics processes. The agent paradigm, in this case, guarantees that patients (through their agents) can establish and negotiate how and what data is collected from them, which data sources can be considered, which data is shared and with whom, or what kind of processing is allowed. In the same way, healthcare professionals may request through their agents, what kinds of data are requested form a patient trajectory, which kind of data analytics are necessary, and what other collaborations or cooperation mechanisms are needed with other physicians, nurses or other personnel.

The main contributions of this work can be summarized as follows: we (i) identify the main challenges for decentralized analytics of patient trajectories (“Challenges in patient trajectories: Modeling and analytics”); (ii) establish a set of design principles of agent interaction models for patient trajectories represented through ontologies (“Patient trajectory agents: Design principles”); (iii) propose a multi-agent architecture that complies with those principles (“Agent-based architecture for patient trajectory management”); and (iv) provide an example of how this approach can be applied in the context of cancer survivor trajectories (“Case study scenario: Trajectories of cancer survivors” and “Cancer survivors support with τ Agents”).

## Case study scenario: Trajectories of cancer survivors

Cancer is one of the main causes of death worldwide, and diagnosed cases are expected to increase significantly in the next decades [9]. Although the different forms of cancer affect a large portion of the population, including millions of patients in working age, recent advances in early detection and treatment are already showing promising results [34]. In Europe, more than 50% of cancer patients survive five years or more after diagnosis, and a number of them are able to return to work and daily life activities, although experiencing side-effects and other conditions due to their treatment [29]. These patients endure different physical and psychological issues after cancer treatment has ceased, potentially during long-term periods. These issues are known to affect the quality of life (QoL) significantly and include reduced physical activity, increased fatigue, fear of cancer recurrence, emotional distress, etc. [24, 38]. Although there is evidence that specific changes in behavior can lead to better outcomes for survivors [21] –e.g., changes in diet, moderate exercise, cognitive therapies– in practice, it is difficult to adapt these recommendations to individual needs, preferences, expectations, and motivation factors.

**Fig. 1:**
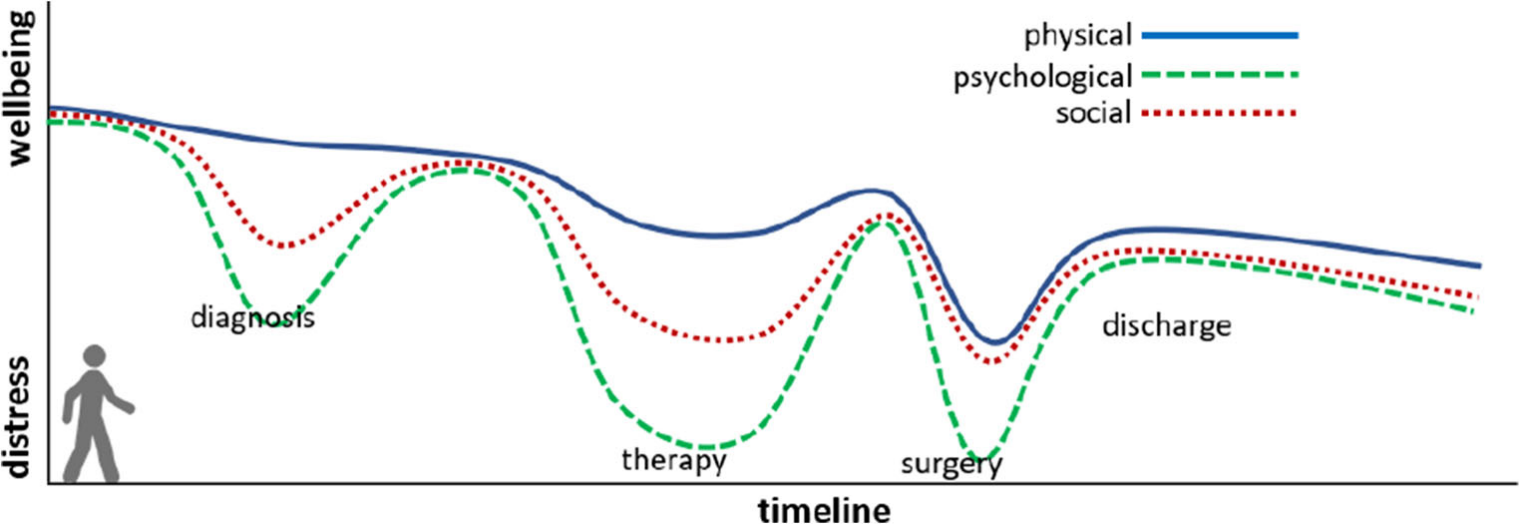
Schematic view of a patient trajectory over time, with respect to general well-being and distress. Notice that the trajectory can be analyzed for different aspects, e.g. physical, psychological, social

Understanding the trajectory of cancer survivors can constitute a fundamental starting point in order to provide useful and personalized suggestions or support [26]. Trajectory information can be acquired from several sources, including the EHR of each patient, self-reported information, behavior questionnaires, or wearable data. Events in the trajectory can be used to identify associations between symptoms, and events, such as therapies, interventions, admissions, re-admissions, etc. (Fig. 1). Trajectories can be used to assess risks as well as to establish predictive models associating symptoms, diseases and outcomes. As we can see in Fig. 1, the trajectory of a patient has a direct incidence not only on her physical well-being but also on the social and psychological aspects of her life. Therefore, the trajectory information can help coping with disease sequels and issues affecting physiological and physical characteristics, while also supporting a broader scope of quality of life aspects.

An additional difficulty for managing cancer survivor trajectories is the need to share data among different institutions and entities, entailing an inherently distributed scenario, while guaranteeing privacy requirements. Survivors are generally at home, and a lot of the information produced at this point is acquired through apps, self-reported outcomes and other instruments. Moreover, EHR data may come from different hospitals and clinics where the patient was treated, e.g. for chemotherapy, physiotherapy, radiotherapy, or surgery, even in different geographical locations. Without coordination mechanisms, the patient is left with the burden of managing her own data, and having to use ad-hoc procedures for sharing it among clinical and medical professionals.

## Challenges in patient trajectories: Modeling and analytics

The modeling of patient trajectories is not straightforward, given the diversity of information sources, and the broad scope of data that they may include, from demographics to physiological or psychological observations. We can summarize these challenges according to the following aspects:

### Trajectory information heterogeneity

A fundamental issue for the modeling of trajectories is related to the vast number of information that can potentially be integrated. Depending on the objectives of the analytics to be performed, trajectories must be able to include different types of data. For example, in Table 1, we identify items form EHR and other sources that could be relevant for the trajectory of a cancer survivor [14, 41]. The degree of heterogeneity requires the usage of models that incorporate semantics, potentially spanning very different aspects: diagnostics, treatments, medication, laboratory, imaging, quality of life, etc.

**Table 1:** Relevant aspects for patient trajectories of cancer survivors from different sources

Aspects	Potential parameters	Source
Demographics	age, gender, marital status, employment, etc.	EHR
General indicators	BMI, weight, height, blood pressure, etc.	EHR +
		Monitoring
Diagnosis	Cancer type, disease stage, tumor location,	EHR
	time after diagnosis, etc.	
Treatment	surgery, ostomy, radiation, chemotherapy, etc.	EHR
Co-morbidities	hypertension, diabetes, CVD, chronic lung disease,	EHR
	high cholesterol	
Symptom burden	fatigue, sleep disturbances, depression, pain,	Self-reported +
	cognitive dysfunction, insomnia	Monitoring
Quality of life	physical, psychological and social functioning	Self-reported

### Patient data sources

Trajectory information may be acquired from different repositories and devices. Models must define interaction mechanisms for acquisition, negotiation, and exchange of trajectory data from heterogeneous sources (see Table 1). For example, cancer survivor data may include retrospective information extracted from EHR records in one or more hospitals and clinics. It may also comprise continuous measures from a wearable device (e.g., for physical activity), or even chatbot interactions and questionnaire responses (e.g., emotional assessment).

### Trajectory data integration & aggregation

In order to analyze trajectories, it is necessary to combine not only different data sources but also from large numbers of patients. Using machine learning or other AI techniques, it is then possible to extract relevant insights, derive patterns, and classify trajectory trends. The acquisition of these data requires protocols for establishing the conditions on which data will be used, how it will be processed, and what outcomes might be obtained.

### Life-long dynamic trajectories

Trajectories can span several years, and may also include live data collected daily (or instantaneously) through sensing devices. Trajectory analysis must be able to cope with this dynamicity and incorporate on-demand analytics that adapts through time and according to the evolution of the patient pathway. For example, trajectory predictions can help dramatically improving quality-of-life indicators in cancer survivors.

### Data analytics explainability

Although AI-based analytics have shown impressive results for classification, prediction, and pattern identification, they often lack in terms of understandability and interpretability. Patient trajectory analytics should be able to provide explainable outcomes, potentially combining and reconciling complementary predictors. In particular, for cancer survivors explanations can lead to stronger motivation and self-efficacy regarding a therapy or treatment.

### Privacy and confidentiality

Given the sensitive nature of trajectory data, privacy has to be guaranteed along the process of acquisition, exchange, processing, and storage. Following current regulations in privacy (e.g., GDPR in the EU), patients’ rights must be respected, e.g., granting access to selected data, accepting or rejecting consent conditions, deleting personal data partially/entirely, or obtaining one’s personal data collections.

## Patient trajectory agents: Design principles

To address the challenges described in “Challenges in patient trajectories: Modeling and analytics”, we propose the representation of trajectories using semantic models and embedding interactions in a multi-agent environment according to the following design principles.

### Ontology-based trajectory modeling

**Fig. 2:**
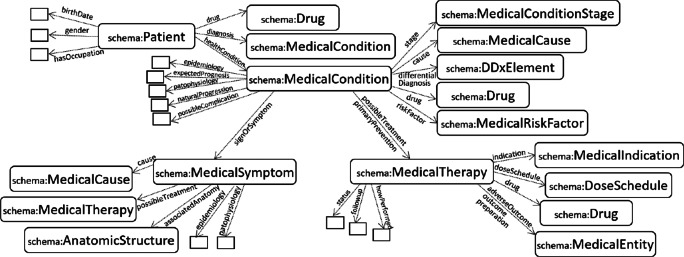
Excerpt from schema.org [22] of relevant medical concepts for patient trajectories. For simplicity, empty boxes represent unspecified types

Our model proposes using ontologies to represent trajectories, as well as connected aspects, including illnesses, admission/discharge events, periodical observations, diagnosis, etc. As a result, trajectories can be represented as knowledge graphs with precise semantics and upon which reasoning and analytics can be applied [6, 7]. The advantages of using ontologies are numerous, as they provide semantics-by-design, allow overcoming heterogeneity, facilitate the interconnection of diverse sources, and can be used as the backbone of logic-based reasoning. In particular, this paper focuses on the use of the widely used schema.org [22] vocabulary (see Fig. 2), which contains a set of medical concepts related to trajectory aspects, including symptoms, medical conditions, therapies, diagnosis, etc.

### Standard semantic vocabularies

**Fig. 3:**
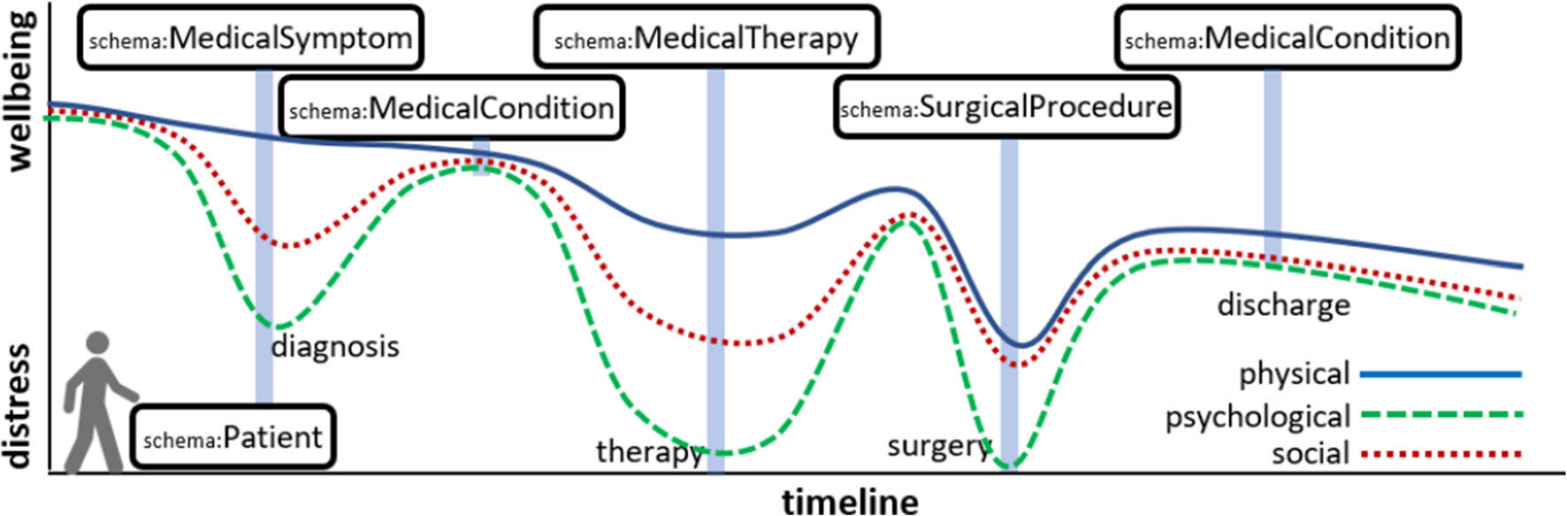
Schematic view of a patient trajectory, aligning with schema.org medical concepts: symptoms, conditions, therapies, surcial procedures, etc

Several ontologies have been standardized, especially in the health domain. These include medication standards, laboratory codes, diagnosis, biomedical concepts, among many others. Moreover, generic health vocabularies, such as the schema.org medical terms, can be used to have a common way of referring to trajectories and their related concepts. Our architecture, as seen later, is based on the use of standard semantic models, i.e., RDF and ontologies in the health domain. As seen in Fig. 2, the popular schema.org vocabulary contains standard terms, which can be complemented with specific medical ontologies like MeSH [32] or ICD-10 [33]. Moreover, as seen in Fig. 3, we can use these terms to represent the different events and stages in the patient trajectory, e.g., symptoms, therapies, surgical procedures, conditions, etc.

### Agent-based entity modeling.

The multi-agent paradigm enables decentralized interactions among entities concerned with patient trajectories. These include the patient itself, which includes her behaviors, goals, and knowledge. Data acquisition processes can also be modeled as agents, coordinating trajectory building with other agents that implement analytics processing, confidentiality negotiation, or aggregation on behalf of a clinical institution (e.g., for a research study). We propose modeling all entities intervening in the generation, processing, and consumption of trajectory information.

### Multi-agent behaviors for trajectory interactions

Interactions among agents managing trajectories can be governed through dynamic behaviors, considering changes that may occur during the period of observation or study. These behaviors may include ML or other AI-based processing of trajectory data; or in a meta-level, the negotiation of exchange of trajectories. Regarding data aggregation, the behavior of an agent representing a clinical study may require managing interactions within a cohort of patients or the request for crowd-sourced data. In all of these, the decentralized nature of these behaviors makes it possible to avoid top-down governance schemes, which are unfeasible in multi-party clinical studies and support environments.

### Negotiation in trajectory processing

The multi-agent paradigm includes the possibility of incorporating negotiation mechanisms at different levels of trajectory analysis. For example, a processing agent using ML techniques may require detailed EHR records for training, which could potentially clash with a patient agent’s goal regarding data anonymity. A negotiation could be established to comply with both parties’ expectations. Other negotiation protocols can be set up, for instance, by coaching agents, which may propose different treatment strategies to a patient agent. A dialogue between the two parties can then be established in order to agree on the most suitable strategy to follow jointly. Our model considers these negotiation patterns a fundamental element in the decentralized management of patient trajectories.

### Personal data privacy interactions

Agents must be designed to comply with existing regulations for data privacy (e.g., GDPR). In this regard, it is fundamental to consider semantic models representing personal data handling concepts, including consent, purpose, processing, legal basis, controllers, and recipients, among others [36]. Agents can, therefore, exchange patient trajectory data, only if consent requirements are met, and according to the legal constraints reflected with these semantic vocabularies.

## Agent-based architecture for patient trajectory management

**Fig. 4:**
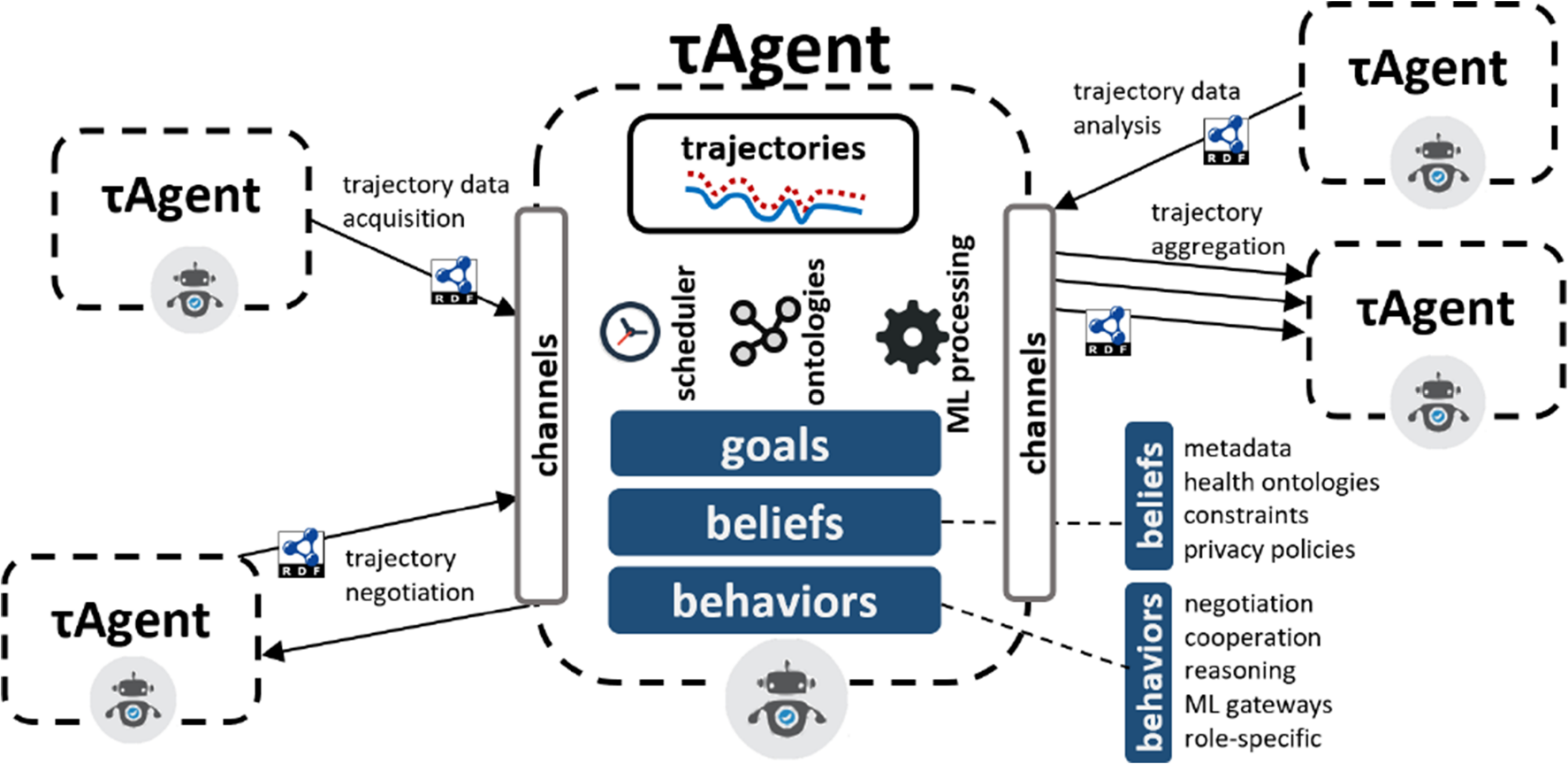
Schematic view of *τ* Agents for managing patient trajectories

This section presents a conceptual architecture of an agent-based approach for patient trajectory management, relying on the use of ontology-driven data models. The central element in this architecture is the *τ* Agent, which s a patient trajectory management agent (Fig. 4). Agents of this type can play different specific roles, such as a patient agent, a processing agent, coaching agent, aggregator agent, and acquisition agent. A *τ* Agent is characterized by a set of goals, beliefs, and behaviors; and includes a specialized knowledge graph of patient trajectory data (partial, complete and/or aggregated). Moreover, it employs a set of channels for communication with other *τ* Agents, a scheduler for establishing task allocation strategies, a set of standard ontologies for trajectory and medical data representation, and (optionally) a set of ML analytics components.

*τ* Agent goals may differ according to the assumed role [39]. For a patient *τ* Agent, the goals may be related, for instance, to quality of life indicators. For example, a goal of an agent acting on behalf of cancer survivor, could be to retain moderate physical activity over a certain period, in order to reduce risk factors of recurrence. Conversely, a coaching agent may define goals regarding the adherence of its assigned patients to their individual treatments or therapies. This could be measured using different indicators, e.g., through quantitative instruments.

**Fig. 5:**
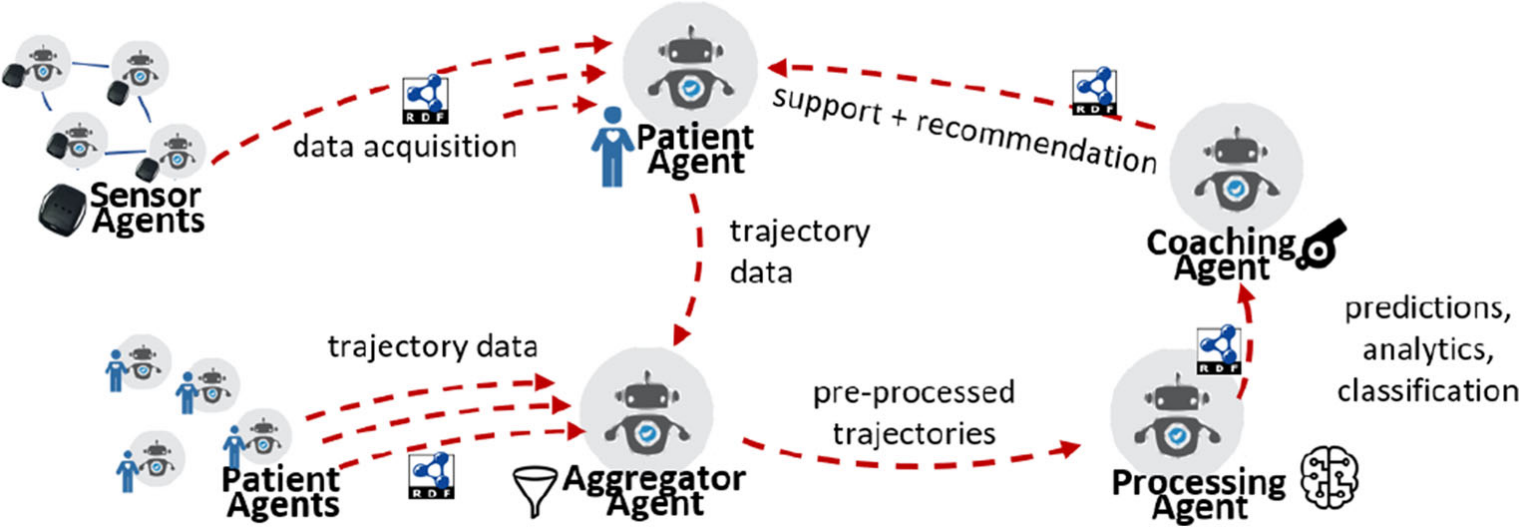
Interactions among *τ* Agents assuming different roles. All interactions rely on the usage of semantic RDF messages

Similarly, beliefs can be defined differently according to the agent role. In general, beliefs include metadata of other agents (e.g., patient agents subscribed to a coaching agent, or potential trajectory contributors for training a ML agent), health vocabularies, constraints, and privacy policies. These beliefs can be crucial later on, for example, during a negotiation among different agents. For instance, a coaching agent belief set can be periodically updated in order to follow the evolution of a patient trajectory, so that future support actions are adapted to the current situation. Behaviors may require access to different functionalities. In the case of processing *τ* Agents, this may include gateways for machine learning methods or reasoning over the trajectory knowledge graphs. All communication channels in *τ* Agents use RDF [16] as underlying representation model (Figs. 4 & 5).

In Fig. 5 we provide a detailed example of interactions among *τ* Agents assuming different roles. A patient agent acting on behalf of a human may solicit data from data acquisition agents, i.e., those gathering data from sensors in the patient environment. Upon negotiation of the data acquisition terms, sensor agents may periodically send data to the patient agent, which can then construct its own trajectory, which will be part of its own beliefs. Then, an aggregator agent may request, through a negotiation protocol, data to several patient agents. To accept or reject this request, the different privacy regulations and preferences, as well as usage and consent information, are fundamental. Patient agents agreeing to aggregate their data, will probably expect further processing to produce actionable feedback. Precisely, a processing agent may then use the aggregated trajectories to create (e.g., prediction) models using ML techniques. The outcomes of the processing of patient trajectories can then be used by a coaching agent to provide support and recommendations to the patients that initially contributed their data.

As can be seen, this conceptual architecture emphasizes on the decentralized nature of patient trajectory interactions. *τ* Agents can respond to entirely different goals, even leading to potential conflicts that would require negotiation to be solved. Moreover, the approach also encourages support for different levels of commitment within the agent environment. This responds to the personalized requirements of patient support systems. For example, cancer survivors may have different levels of adherence to treatment and very different illness pathways.

**Fig. 6:**
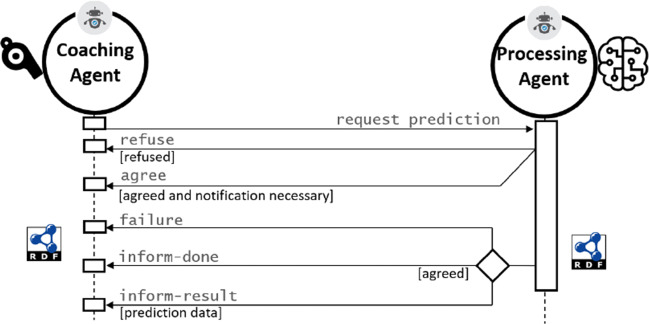
*τ* Agent interaction following the FIPA request interaction protocol

Interactions among *τ* Agents can be embedded in standard agent protocols such as FIPA [1]. For example, as seen in Fig. 6, a coaching agent may require prediction results from a processing agent, regarding potential outcomes of a given patient. This request can be encoded as a Request Interaction Protocol, to which the processing agent may agree or refuse. In case of acceptance, the prediction data can be transmitted. All interactions are encoded in RDF in the proposed architecture.

## Cancer survivors support with *τ* Agents

To illustrate the different interactions among *τ* Agents, we present excerpts of semantically annotated data representing excerpts and parts of patient trajectories, for the case scenario of colorectal cancer survivors.

Consider a patient who has survived colon cancer and is now following a long-life support program. His patient agent is in charge of managing his patient trajectory, and for this purpose, it collects EHR information available from agents representing the different hospitals and clinics where he was treated. Moreover, and assuming that the support program includes the usage of wearable devices that monitor physical activity, stress, and behavior, the patient trajectory can be completed with live data integrated continuously.

**Listing 1:**
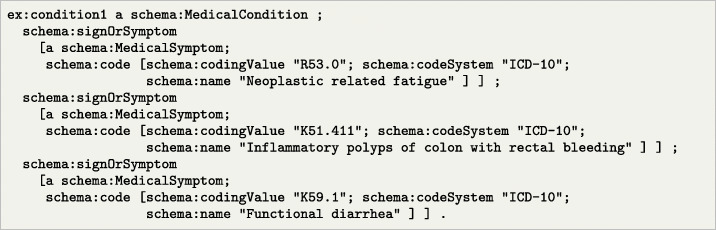
Example of symptoms encoded with ICD-10 and following schema.org represented in RDF Turtle format. All prefixes omitted for brevity

**Listing 2:**
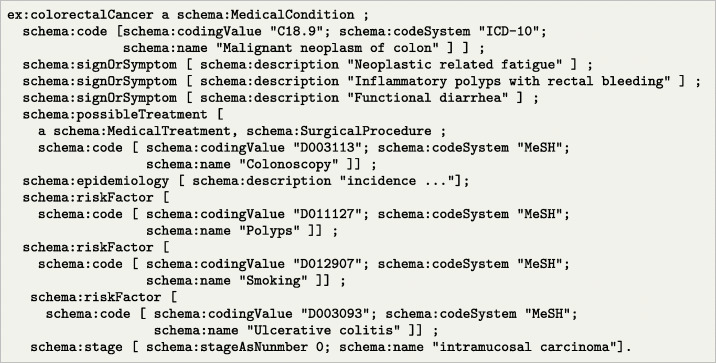
Example of colorectal cancer details described with schema.org

**Listing 3:**
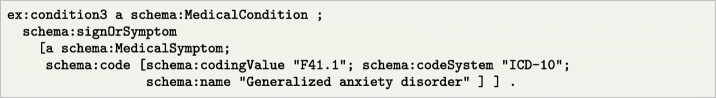
Example of a medical condition –anxiety– for a cancer survivor

**Listing 4:**
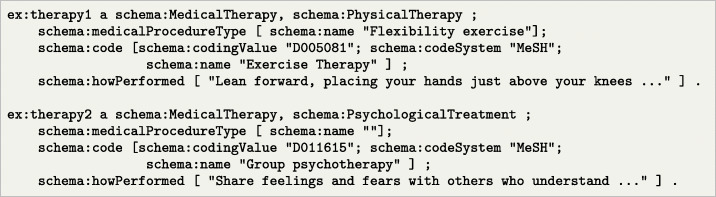
Example of potential therapies for a cancer survivor –flexibility exercises and psychological group therapy

In Listing 1, we illustrate how we can represent a set of symptoms from a patient, using the schema.org vocabulary. In the example, the patient symptoms are encoded as MedicalSymptom instances, with codes referring to a specific medical coding system (in this case, the ICD-10 standard). These symptoms, i.e., fatigue, rectal bleeding, and diarrhea, can be integrated as part of the patient trajectory and could be used later for stratification or classification.

The symptomatic and diagnosis information is only one small part of the patient trajectory. Additional information can be appended, including the colon cancer diagnose itself (Listing 2), treatments such as a colonoscopy, epidemiology, risk factors, stage of cancer, etc. Many of these pieces of information can be used in different ways during a support program. Just as an example, considering that risk factors such as polyps or smoking habits can be linked to future recurrence of cancer, the coaching agent may choose to propose actions that reduce those risks. Notice that we can use different coding systems, as in the case of risk factors, where the MeSH [32] standard is employed.

Furthermore, during the program, a cancer survivor may suffer not only from physical problems but also from psychological issues. As an example, consider that the patient suffers from anxiety, mainly due to the fact of having fear of recurrence. Using a self-reported questionnaire (e.g., through a mobile app), or supported by wearable devices that compute stress levels, and anxiety symptom can be established, encoded with ICD-10 in Listing 3.

Having this information, the coaching agent can propose actions, in this case potential therapies and activities that could help the patient dealing with his conditions. As an example, in Listing 4 we include both an exercise therapy (flexibility) and psychological therapy (group psychotherapy).

## Discussion and related work

The proposed conceptual architecture is based on two fundamental ideas: (i) the use of semantic representation models, and (ii) the multi-agent paradigm. Both show complementary properties allowing the establishment of decentralized networks of potentially independent agents, which can establish cooperation and negotiation mechanisms to achieve their goals. Although at this stage, the proposed model does not materialize into an implementation, it already establishes the main guiding principles that should be observed. In particular, we can emphasize on the *τ* Agent basic structure, the types of roles that can be implemented, the usage of RDF for inter-agent communication, the reliance on standard vocabularies such as schema.org, and of medical ontologies like ICD-10 or MeSH. We believe that this approach can lead to promising results, especially for use-cases where patient trajectories can be exploited using large volumes of data while maintaining personal data preferences and guarantees. We identify several aspects in which further research is required in order to address the challenges identified above, and we relate them to existing work in the literature.

### Ontology agreement

Matching terms among ontologies is a long-studied topic, and in this case, it will be necessary to align concepts from different vocabularies, and even data models [25]. For example, patient trajectories could be specified both using schema.org and the FHIR^1^ specifications. Moreover, a large number of medical specific codes can make it hard to overcome potential coding discrepancies. Several works in the literature have used ontology-based approaches for health data integration [17, 30]. However, only few works include the modeling of interactions, negotiation, and collaboration among intelligent and autonomous systems [11], as in *τ* Agents.

### Agent autonomy

We presented different profiles for *τ* Agents, including specialized sensor data acquisition agents. Nevertheless, given that it is often the case that sensing and wearable devices have limited computation capabilities, it becomes challenging to deploy intelligent agents on such platforms. Although there have been recent proposals on how to adapt multi-agent systems to these environments, e.g., incorporating real-time support [12] or scheduling strategies [13], the integration of these data into semantic trajectories remains to be implemented.

### Implementation

The implementation of the proposed agent-based model is one of the key aspects to consider in the immediate future. This implementation will need to consider the communication interactions as described earlier in the paper and using ontologies such as schema.org as a first-class citizen. Nevertheless, given the open nature of semantic vocabularies, it is at the same time advantageous for extensibility purposes, but problematic as the number of models to integrate can be incompatible or hard to align. The implementation will also consider the issues of agent discovery, negotiation implementation, and publishing patient trajectories. Previous works have explored the integration of health agents through semantic services [11] and ontology-based approaches [23, 40], although lacking the concept of patient trajectories.

### Recommendation & support

The proposed architecture serves as a platform for eHealth support. Therefore, the high-level challenge is to provide useful recommendations and advice. We plan to implement the use-case for cancer survivors, following the principles and examples shown in this paper. Beyond existing works in the area, including eHealth support and Semantic Web architectures for patient support [5, 23], we combine both the modelling of trajectories and of agents’ behaviors. An additional challenge will be to effectively assess the adequacy and accuracy of the recommendation with respect to the survivors’ needs, goals, and expectations.

### Explainability

A general challenge regarding data analytics, and especially when using ML techniques, is explainability. This is even more important in eHealth, where decisions can have vital consequences. In this case, future work should also consider not only the of symbolic knowledge from ML predictors but also the integration of heterogeneous knowledge and negotiation among explainability agents [15]. Agents may need to have reliable explanations of analysis and decisions taken regarding a trajectory, before choosing a behavior change strategy [2].

### Evaluation and validation

Several indicators must be considered for evaluation of this approach, including not only performance metrics for communication and decision making but also considering the effectiveness of negotiations, accuracy of data analytics, response time of agent interactions, compliance to privacy policies, etc. While a number of ontology-based medical system have been evaluated in the last decade [28, 35, 37, 40], the incorporation of trajectory and agent-based modelling requires a thorough assessment, e.g. by running pilot studies.

## Conclusions

In this paper, we presented a novel approach based on multi-agent systems for managing patient trajectories, which are represented and exchanged using semantic models. We identified first a set of challenges in this context, for which we proposed a corresponding set of design principles. In turn, these principles guide our proposal for a conceptual architecture that defined what we call *τ* Agents, which can assume different roles. Furthermore, we exemplified how this architecture can be used to acquire patient trajectory data, aggregate them, and apply AI algorithms to provide input for coaching agents. The entire concept has been used to illustrate a concrete use-case, i.e., for cancer survivorship support. Finally, we have proposed a research agenda that continues addressing the different challenges described in the paper, targeting not only scientific but also societal impact through the development of decentralized eHealth applications.

## Footnotes

1 http://hl7.org/fhir

## References

[1] Foundation for Intelligent Physical Agents Standard. http://www.fipa.org/

[2] Abdulrahman A., Richards D., Ranjbartabar H., Mascarenhas S.: Belief-based agent explanations to encourage behaviour change.. In: Proceedings of the 19th ACM International Conference on Intelligent Virtual Agents, 2019, pp 176–178

[3] Alexander GL (2007). The nurse—patient trajectory framework. Studies in Health Technology and Informatics.

[4] Ardissono L, Goy A, Petrone G, Segnan M (2005). A multi-agent infrastructure for developing personalized web-based systems. ACM Trans. Internet Technol. (TOIT).

[5] Benyahia A.A., Hajjam A., Hilaire V., Hajjam M.: e-care: Ontological architecture for telemonitoring and alerts detection.. In: 2012 IEEE 24Th International Conference on Tools with Artificial Intelligence, vol 2. IEEE, 2012, pp 13–17

[6] Berners-Lee T, Bizer C, Heath T (2009). Linked data-the story so far. IJSWIS.

[7] Berners-Lee T, Hendler J, Lassila O (2001). The semantic Web. Scientific American.

[8] Blumenthal D, Tavenner M (2010). The “meaningful use” regulation for electronic health records. New England J. Medicine.

[9] Bray F, Ferlay J, Soerjomataram I, Siegel RL, Torre LA, Jemal A (2018). Global cancer statistics 2018: Globocan estimates of incidence and mortality worldwide for 36 cancers in 185 countries. CA:, a Cancer Journal for Clinicians.

[10] Buonocunto P, Giantomassi A, Marinoni M, Calvaresi D, Buttazzo G (2018). A limb tracking platform for tele-rehabilitation. ACM Trans. Cyber. Phys. Syst..

[11] Caceres C, Fernández A, Ossowski S, Vasirani M (2006). Agent-based semantic service discovery for healthcare: an organizational approach. IEEE Intel. Syst..

[12] Calvaresi D, Cesarini D, Sernani P, Marinoni M, Dragoni AF, Sturm A (2017). Exploring the ambient assisted living domain: a systematic review. J. Amb. Intel. Humanized Comput..

[13] Calvaresi D., Marinoni M., Lustrissimini L., Appoggetti K., Sernani P., Dragoni A.F., Schumacher M., Buttazzo G.: Local scheduling in multi-agent systems: getting ready for safety-critical scenarios.. In: Multi-agent Systems and Agreement Technologies. Springer, 2017, pp 96–111

[14] Chung JY, Lee DH, Park JH, Lee MK, Kang DW, Min J, Kim DI, Jeong DH, Kim NK, Meyerhardt JA (2013). Patterns of physical activity participation across the cancer trajectory in colorectal cancer survivors. Supportive Care in Cancer.

[15] Ciatto G., Calegari R., Omicini A., Calvaresi D.: Towards XMAS: explainability through multi-agent systems.. In: Proceedings of the 1st Workshop on Artificial Intelligence and Internet of Things co-located with AI*IA 2019, 2019, pp 40–53

[16] Cyganiak R., Wood D., Lanthaler M. (2014) Rdf 1.1 concepts and abstract syntax. W3c Recommendation 25(02). https://www.w3.org/TR/rdf11-concepts/

[17] Dimitrieski V., Petrović G., Kovačević A., Luković I., Fujita H.: A survey on ontologies and ontology alignment approaches in healthcare.. In: International Conference on Industrial, Engineering and Other Applications of Applied Intelligent Systems. Springer, 2016, pp 373–385

[18] Dubovitskaya A., Urovi V., Barba I., Aberer K., Schumacher M.I. (2016) A multiagent system for dynamic data aggregation in medical research. BioMed Research International 2016 10.1155/2016/9027457 PMC5128729 27975063

[19] Eslami MZ, Zarghami A, Sapkota B, Van Sinderen M (2010). Service tailoring: Towards personalized homecare services. ACT4SOC.

[20] Falcionelli N, Sernani P, Brugués A, Mekuria DN, Calvaresi D, Schumacher M, Dragoni AF, Bromuri S (2019). Indexing the event calculus: towards practical human-readable personal health systems. Artific. Intel. Medic..

[21] Finne E, Glausch M, Exner AK, Sauzet O, Stoelzel F, Seidel N (2018). Behavior change techniques for increasing physical activity in cancer survivors: a systematic review and meta-analysis of randomized controlled trials. Cancer Manage. Res..

[22] Guha RV, Brickley D, Macbeth S (2016). Schema. org: evolution of structured data on the web. Commun. ACM.

[23] Hussain S., Abidi S.R., Abidi S.S.R.: Semantic web framework for knowledge-centric clinical decision support systems.. In: Conference on Artificial Intelligence in Medicine in Europe. Springer, 2007, pp 451–455

[24] Jones JM, Olson K, Catton P, Catton CN, Fleshner NE, Krzyzanowska MK, McCready DR, Wong RK, Jiang H, Howell D (2016). Cancer-related fatigue and associated disability in post-treatment cancer survivors. Journal of Cancer Survivorship.

[25] Khan WA, Khattak AM, Hussain M, Amin MB, Afzal M, Nugent C, Lee S (2014). An adaptive semantic based mediation system for data interoperability among health information systems. J. Med. Syst..

[26] Klimmek R., Wenzel J.: Adaptation of the illness trajectory theory to describe the work of transitional cancer survivorship.. In: Oncology Nursing Forum, vol 39. NIH Public Access, 2012, p e499 10.1188/12.ONF.E499-E510 PMC3664233 23107863

[27] Koutkias VG, Chouvarda I, Triantafyllidis A, Malousi A, Giaglis GD, Maglaveras N (2009). A personalized framework for medication treatment management in chronic care. IEEE Trans. Inform. Technol. Biomed..

[28] Lasierra N, Roldán F, Alesanco A, García J (2014). Towards improving usage and management of supplies in healthcare: An ontology-based solution for sharing knowledge. Expert Systems with Applications.

[29] Liu L, O’Donnell P, Sullivan R, Katalinic A, Moser EC, de Boer A, Meunier F, Scientific Committee O (2016). Cancer in europe: Death sentence or life sentence?. European Journal of Cancer.

[30] Liyanage H, Krause P, de Lusignan S (2015). Using ontologies to improve semantic interoperability in health data. BMJ Health & Care Informatics.

[31] Murray SA, Kendall M, Boyd K, Sheikh A (2005). Illness trajectories and palliative care. Bmj.

[32] Nelson S.J., Schopen M., Savage A.G., Schulman J.L.A., Arluk N.: The mesh translation maintenance system: structure, interface design, and implementation.. In: Medinfo, 2004, pp 67–69 15360776

[33] Organization W.H., et al. (2004) Icd-10: international statistical classification of diseases and related health problems: tenth revision 3376487

[34] Organization W.H., et al. (2017) Guide to cancer early diagnosis

[35] Paganelli F, Giuli D (2010). An ontology-based system for context-aware and configurable services to support home-based continuous care. IEEE Trans. Inform. Technol. Biomed..

[36] Pandit H.J., Polleres A., Bos B., Brennan R., Bruegger B., Ekaputra F.J., Fernández J. D., Hamed R.G., Kiesling E., Lizar M., et al.: Creating a vocabulary for data privacy.. In: OTM Confederated International Conferences on the Move to Meaningful Internet Systems. Springer, 2019, pp 714–730

[37] Parry D (2006). Evaluation of a fuzzy ontology-based medical information system. International Journal of Healthcare Information Systems and Informatics.

[38] Van Leeuwen M, Husson O, Alberti P, Arraras JI, Chinot OL, Costantini A, Darlington AS, Dirven L, Eichler M, Hammerlid EB (2018). Understanding the quality of life issues in survivors of cancer: towards the development of an eortc qol cancer survivorship questionnaire. Health and Quality of life Outcomes.

[39] Vermunt NP, Harmsen M, Westert GP, Rikkert MGO, Faber MJ (2017). Collaborative goal setting with elderly patients with chronic disease or multimorbidity: a systematic review. BMC Geriatrics.

[40] Wang MH, Lee CS, Hsieh KL, Hsu CY, Acampora G, Chang CC (2010). Ontology-based multi-agents for intelligent healthcare applications. Journal of Ambient Intelligence and Humanized Computing.

[41] Wu HS, Harden JK (2015). Symptom burden and quality of life in survivorship: a review of the literature. Cancer Nursing.

